# CD14^+^ macrophages that accumulate in the colon of African AIDS patients express pro-inflammatory cytokines and are responsive to lipopolysaccharide

**DOI:** 10.1186/s12879-015-1176-5

**Published:** 2015-10-17

**Authors:** Edana Cassol, Theresa Rossouw, Susan Malfeld, Phetole Mahasha, Tomas Slavik, Chris Seebregts, Robert Bond, Johannie du Plessis, Carl Janssen, Tania Roskams, Frederik Nevens, Massimo Alfano, Guido Poli, Schalk W. van der Merwe

**Affiliations:** MRC Unit for Inflammation and Immunity, Department of Immunology and the Tshwane Academic Division of the National Health Laboratory Service, University of Pretoria, Pretoria, South Africa; Department of Health Sciences, Carleton University, 5433 Herzberg Laboratories, 1125 Colonel By Drive, Ottawa, Ontario K1S 5B6 Canada; Department of Family Medicine, University of Pretoria, Pretoria, South Africa; Department of Anatomical Pathology, University of Pretoria and Ampath Pathology Laboratories, Pretoria, South Africa; Jembi Health Systems NPC, Durban, South Africa; School of Mathematics, Statistics and Computer Science, University of KwaZulu-Natal, Durban, South Africa; Hepatology and GI-Research Laboratory, University of Pretoria, Pretoria, South Africa; Translational Cell and Tissue Research, Department of Imaging and Pathology, University of Leuven, Leuven, Belgium; Department of Hepatology, University of Leuven, Leuven, Belgium; San Raffaele Scientific Institute, School of Medicine, Milan, Italy; Vita-Salute San Raffaele University, School of Medicine, Milan, Italy; Institute of Human Virology, University of Maryland School of Medicine, Baltimore, MD USA; Department of Internal Medicine, Division of Liver and Biliopancreatic Disorders, University of Leuven, Leuven, Belgium; Present Address: Division of Experimental Oncology/Unit of Urology, URI, IRCCS Ospedale San Raffaele, Milan, Italy

**Keywords:** AIDS, Macrophages, Inflammation, Immune activation, Colon

## Abstract

**Background:**

Intestinal macrophages are key regulators of inflammatory responses to the gut microbiome and play a central role in maintaining tissue homeostasis and epithelial integrity. However, little is known about the role of these cells in HIV infection, a disease fuelled by intestinal inflammation, a loss of epithelial barrier function and increased microbial translocation (MT).

**Methods:**

Phenotypic and functional characterization of intestinal macrophages was performed for 23 African AIDS patients with chronic diarrhea and/or weight loss and 11 HIV-negative Africans with and without inflammatory bowel disease (IBD). AIDS patients were treated with cotrimoxazole for the prevention of opportunistic infections (OIs). Macrophage phenotype was assessed by flow cytometry and immuno-histochemistry (IHC); production of proinflammatory mediators by IHC and Qiagen PCR Arrays; in vitro secretion of cytokines by the Bio-Plex Suspension Array System. Statistical analyses were performed using Spearman’s correlation and Wilcoxon matched-pair tests. Results between groups were analyzed using the Kruskal-Wallis with Dunn’s post-test and the Mann–Whitney U tests.

**Results:**

None of the study participants had evidence of enteric co-infections as assessed by stool analysis and histology. Compared to healthy HIV-negative controls, the colon of AIDS patients was highly inflamed with increased infiltration of inflammatory cells and increased mRNA expression of proinflammatory cytokine (tumour necrosis factor (TNF)-α, interleukin (IL)-1β, IFN-γ, and IL-18), chemokines (chemokine (C-C motif) ligand (CCL)2 and chemokine (C-X-C) motif ligand (CXCL)10) and transcription factors (TNF receptor-associated factor (TRAF)6 and T-box (TXB)21). IHC revealed significant co-localization of TNF-α and IL-1β with CD68^+^ cells. As in IBD, HIV was associated with a marked increase in macrophages expressing innate response receptors including CD14, the co-receptor for lipopolysaccharide (LPS). The frequency of CD14^+^ macrophages correlated positively with plasma LPS, a marker of MT. Total unfractionated mucosal mononuclear cells (MMC) isolated from the colon of AIDS patients, but not MMC depleted of CD14^+^ cells, secreted increased levels of proinflammatory cytokines ex vivo in response to LPS.

**Conclusions:**

Intestinal macrophages, in the absence of overt OIs, play an important role in driving persistent inflammation in HIV patients with late-stage disease and diarrhea. These results suggest intensified treatment strategies that target inflammatory processes in intestinal macrophages may be highly beneficial in restoring the epithelial barrier and limiting MT in HIV-infected patients.

## Background

The human immunodeficiency virus type (HIV) induces rapid and profound damage to the gut-associated lymphoid tissue (GALT). Damage includes an early massive depletion of the T helper (Th)17 subset of CCR5^+^CD4^+^ memory T cells [1–7], changes in the frequency of CD4^+^CD25^+^FoxP3^+^ T regulatory (Treg) cells [8–10], an influx of activated cytotoxic CD8^+^ T lymphocytes [11], irreversible activation-induced fibrosis [12, 13] and a loss of epithelial integrity leading to increased microbial translocation (MT) [14]. MT is a major cause of local and systemic immune activation in HIV-infected patients, both before and during antiretroviral therapy (ART) [14–17].

Clinical manifestations of HIV infection in the gastrointestinal tract include malabsorption, diarrhea and a wasting syndrome [18–21]. Diarrhea accompanied by wasting (“slims disease”) is responsible for a significant amount of HIV-related morbidity in sub-Saharan Africa [22], even in the era of ART [23, 24]. Opportunistic infections (OIs) are a common cause of these disorders in HIV-infected individuals and include a range of viral, bacterial, fungal and parasitic pathogens [24]. Cotrimoxazole prophylaxis is a mainstay for the prevention of OIs in Africa and other resource limited settings and is recommended for severely immunocompromised HIV-infected individuals [25]. Prophylactic treatment with cotrimoxazole has been shown to improve survival and reduce morbidity in HIV patients prior to the initiation of ART [25–28].

Intestinal macrophages play a pivotal role, not only in the recognition and elimination of invading pathogens, but also in the regulation of inflammatory responses, maintaining the integrity of the epithelial barrier and in tissue remodeling and repair [29–33]. Under homeostatic conditions, stromal factors such as transforming growth factor-β (TGF-β), induce intestinal macrophages to differentiate into “inflammation anergic” cells that promote tolerance by maintaining the expression of Foxp3 in suppressive Treg cells [34]. These “resident” macrophages have avid phagocytic and bacteriocidal activity but do not express innate response receptors or co-stimulatory molecules and do not produce inflammatory cytokines in response to microbial stimulation [29, 35]. Inflammation anergy plays a central role in preventing damage to the gastrointestinal tract, an organ that is constantly exposed to a large number of commensal bacteria and other antigenic stimuli.

However, under conditions of chronic inflammation, intestinal macrophages can develop a pro-inflammatory phenotype and secrete inflammatory mediators such as TNF-α, interleukin-Iβ (IL-1β), interleukin-6 (IL6), and nitric oxide in response to invading pathogens and antigenic stimuli. Failure to downregulate this inflammatory response, as observed in patients with IBD and cirrhosis, is a major driver of intestinal disease [29, 36, 37]. Pro-inflammatory macrophages in the colon of patients with Crohn’s disease express CD14, produce inflammatory mediators in response to Toll-Like Receptor (TLR) stimulation and contribute to chronic intestinal inflammation via induction of the interleukin-23/interferon-γ (IL-23/IFN-γ)–positive feedback loop [29]. The accumulation of CD14^+^ macrophages in patients with Crohn’s disease has been attributed to an increased recruitment of inflammatory monocytes and a concomitant disruption of physiological, tissue-specific macrophage differentiation [38]. Consistent with these findings, a recent study of the duodenum has shown that HIV infection is associated with increased production of macrophage-related pro-inflammatory molecules (IL-1β, CCL5, CXCL9, and CXCL10) and enrichment of macrophages with low phagocytic activity [39].

In this study, we evaluated the inflammatory *milieu* and characterized the phenotypic and functional properties of macrophages in the colon of cotrimoxazole-treated ART-naïve African AIDS patients with unexplained diarrhea and/or weight loss who had no evidence of a confounding co-infection. Results were compared to those obtained in HIV-negative African controls with and without IBD. We detected a marked increase in pro-inflammatory macrophages in the colon of AIDS patients compared to healthy HIV seronegative controls. These cells expressed CD14 and other innate response receptors and produced inflammatory cytokines (TNF-α and IL-6), both constitutively and in response to LPS stimulation.

## Methods

### Study participants

The initial study cohort consisted of 23 cotrimoxazole-treated, ART-antiretroviral therapy naïve AIDS patients who were undergoing diagnostic endoscopy for unexplained diarrhea (>12 weeks duration) and weight loss (>10 % of the patient’s body weight). Five additional HIV-infected patients were subsequently sampled for ex vivo studies of LPS stimulation and cytokine/chemokine production. All patients had been receiving on Cotrimoxazole for >6 months. After obtaining written informed consent, biopsies were collected from the ascending and descending colon using a standard colonoscope and were processed for flow cytometry and histological evaluation. Blood samples were collected for routine patient management [CD4^+^ and CD8^+^ T cell counts, plasma viral load (VL)] and for quantification of plasma LPS. Patients were eligible for the study if they tested negative for *Mycobacterium tuberculosis* and for enteric pathogens in stool specimens. Subjects with secondary gastrointestinal infections, as determined by culture and microscopic examination of stool specimens and by histological evaluation of biopsies were excluded from the study. *Clostridium difficile* was excluded using three different methods - enzyme immunoassay for toxins A and B, PCR and histopathology. Healthy HIV-seronegative South Africans (*n* = 5) who tested pathogen negative and showed no evidence of disease during screening for colorectal cancer served as controls. These subjects were matched to HIV subjects by age and gender (median age 34, range 26–45 years; 60 % male). Six HIV-negative South Africans with inflammatory bowel disease (IBD) and symptoms of disease activity including abdominal cramps, diarrhea or bloody stools were also recruited. None of the IBD patients had received corticosteroids, azathioprine, 6-Mercaptopurine (6-MP) or anti-TNF therapy during the 6 months prior to enrollment. Following informed consent, biopsies were obtained from areas of active colitis. The study was reviewed and approved by the Research Ethics Committee of the Faculty of Health Sciences, University of Pretoria.

### Specimen collection and processing

Biopsies were collected prior to ART. A total of 8–10 “pinch biopsies” were collected from each patient. Biopsies were placed on ice in tissue culture medium RPMI 1640 supplemented with 10 % fetal calf serum (FCS) and immediately processed for flow cytometry or in vitro stimulations. Biopsies for immunohistochemistry (IHC) were fixed in 10 % neutral buffered formalin and embedded in paraffin; biopsies for PCR array were snap frozen in liquid nitrogen and stored at −80 °C.

### Histological analyses

Hematoxylin and eosin (H&E), periodic acid-Schiff diastase (PAS-d), Ziehl-Neelsen and Giemsa staining were performed to exclude acid fast bacilli (AFB), fungal and parasitic organisms (including Cryptosporidium and Isopora, spp., as well as Microsporidia). Cytomegalovirus, adenovirus and herpes simplex virus were excluded by careful scrutiny of H&E slides supplemented with appropriate IHC staining. Clostridial infection was excluded on morphologic grounds by means of careful evaluation of H&E sections. For T cell quantification, tissue sections were incubated with murine anti-human CD4 (clone 4B12, DAKO, Denmark) or CD8 (clone CD8/CD144B, DAKO, Denmark) monoclonal antibodies (mAbs), visualized with 3,3′-Diaminobenzidine (DAB) and counter-stained with haematoxylin. CD68 (KP1, DAKO, Denmark), CD14 (M0825 clone TUK4, DAKO), IL-1β (Abcam ab8320), IL-6 (Abcam ab9324) and TNF (PeproTech 500-M26) staining was performed on sections retrieved in PT101 Link Pre-Treatment Module (Dako), as previously described [37]. Following blocking of endogenous peroxidase and incubation with rabbit polyclonal anti-CD14 Ab or with mouse mAbs directed against CD68, IL1β, IL-6 or TNF-α, tissue sections were treated with Envision™/Horseradish peroxidase (HRP) dual link polymer, visualized with DAB^+^ chromogen and counter-stained with hematoxylin. IHC staining on two colonic biopsies per patient was evaluated, with the area on the slides showing the most positivity used for evaluation. The number of positive cells in 5 contiguous high power fields (HPF) of 0.80 mm^2^ (magnification x 400) was counted and reported as the average number of macrophages/HPF. Images were acquired, recorded and processed using a Leica DMLB 11888011 microscope and digital camera and an IM50 Image Manager (Leica Microsystems Wetzlar, GmbH, Germany). To determine if CD68^+^ macrophages were producing inflammatory cytokines, colonic biopsies were subjected to sequential double labeling with Abs directed against CD68 and either IL1β or TNF-α using established methods [37]. Slides were incubated with anti-human IL1β or TNF-α primary Ab, washed and incubated with Alexa Fluor-488-conjugated secondary Ab (Invitrogen). After boiling and treatment with 3 % H_2_O_2_/methanol to block the antigenicity of the first set of Abs, the staining procedure was repeated using mouse anti-human CD68 and Alexa Fluor-568-conjugated secondary Ab (Invitrogen). Nuclei were stained with DAPI. The optimal concentration of each primary antibody (either mouse anti-IL1β or anti-TNF-α, or in the case of the second primer set, mouse anti-CD68) was determined using positive (tissue sections from patients with acute intestinal inflammation) and negative (reagents lacking primary antibody) controls and a dilution series of the primary antibody of interest. Data acquisition was performed using a Zeiss Axioplan 2 fluorescent microscope equipped with a triple filter, Fluoarc fluorescent light source and Axiocam ICc1 camera. All studies were performed in a blinded fashion by a histopathologist with extensive experience in the analysis of intestinal specimens.

### Phenotyping of Mucosal Mononuclear Cells (MMC)

MMC were obtained by digesting the biopsies in RPMI 1640 containing 10 % FCS and 0.5 mg/ml collagenase type IV (Sigma, St Louis, MO) for 30 min at 37 °C as previously described [40]. The resultant single cell suspensions were passed through a 70-μm cell strainer to remove debris (Becton Dickenson, Labware, NJ). The remaining tissue fragments were re-digested and pooled digests were strained, washed and processed for flow cytometry or LPS stimulation. Multi-parameter flow cytometry was performed on digested colonic MMC (0.5 x10^6^ cells/tube) that had been stained for 30 min at 4 °C with various combinations of anti-human CD3, CD4, CD8, CD33, CD14, CD16, CD80, and CD86 mAbs (Beckman Coulter Inc., Fullerton, CA). T cell populations were identified based on forward and side-scatter characteristics and expression of CD3. Macrophage populations were identified based on forward and side scatter characteristics and the expression of CD33. CD14, CD16, CD80 and CD86 levels were determined by gating on CD33+ cells. CD33^+^ cells were confirmed to be positive for plasma membrane CD45 and CD68 by back gating during the initial establishment of flow cytometry protocols. Samples were analyzed on a Beckman Coulter FC500 flow cytometer with a minimum of 5000 gated events collected per tube. Results were analyzed using Flow Jo (Tree Star, Ashland, OR).

### PCR array

Expression levels of 84 inflammatory genes were determined using the human Th17 Autoimmunity and Inflammation RT^2^ Profiler PCR array (SABioscience, Frederick, MD). Total RNA was extracted from 12 to 15 mg of colonic tissue using the Maxwell® 16 Tissue LEV Total RNA Purification Kit. cDNA was synthesized from 0.5 μg of total RNA using the RT^2^ PCR array first strand kit. Real time PCR was performed in 96-well plates using RT^2^ SYBR Green qPCR Master Mix and a CFX96 Real Time PCR Detection System (BioRad, Hercules, CA). Ten AIDS patients, 6 IBD patients and 5 HIV negative controls were analyzed in triplicate. Results were normalized using 5 housekeeping genes and analyzed by the comparative cycle threshold method.

### Quantification of plasma LPS

Peripheral venous blood was collected in EDTA tubes at the time of colonoscopy. Plasma was isolated by centrifugation at 1800 rpm for 10 min at 4 °C and stored in 1.0 mL aliquots at −80 °C. LPS levels were quantified using the Limulus Amoebocyte Lysate assay QCL-1000 (Lonza, Valais Switzerland) and expressed as EU/mL as previously described [15].

### LPS stimulation of CD14^+^ and CD14^−^ MMC

CD14^+^ macrophages were depleted from colonic MMC by positive selection (CD14 Microbeads, Miltenyi Biotech). Total MMC and CD14^−^ MMC (0.25x10^6^ cells/well) were cultured in triplicate in RPMI/10 % FCS/5 % human AB serum in the presence or absence of LPS (1 μg/mL; Sigma) for 18 h. Cytokine and chemokine levels (pg/ml) in culture supernatants were quantified using the Bio-Plex Suspension Array System according to manufacturer’s instructions (Bio-Rad).

### Statistical analyses

Prism 5 from GraphPad Software (La Jolla, CA) was used for statistical analyses. Paired observations were compared using Wilcoxon matched pair tests. The Kruskal-Wallis with Dunn’s post-test or Mann–Whitney U tests were used to compare between groups. Linear correlations were assessed using Spearman’s rank correlation coefficient. Two-tailed *p* values <0.05 were considered significant. PCR array fold change calculations were performed using the RT^2^ Profiler PCR Array Data Analysis Template v3.2 (SABioscience).

## Results

### Patient characteristics

Clinical characteristics of the AIDS study cohort (*n* = 23) are shown in Table 1. All patients had high plasma VLs (median 5.08 (4.2–6.6) log_10_ HIV RNA copies/mL) and low blood CD4^+^ T cells counts (median 69 (6–237) cells/uL). Symptoms leading to endoscopy were chronic diarrhea in 13 (56 %) and/or unexplained weight loss in 10 (44 %) patients. Five additional AIDS patients were subjected to ex vivo stimulation studies. None of the 28 patients had evidence of enteric co-infections as assessed by stool analysis and histology. HIV-negative healthy controls (*n* = 5) showed no evidence of structural damage or inflammatory cell infiltrates. HIV-negative IBD patients had typical well-documented Crohn’s disease (CD; *n* = 3), or ulcerative colitis (UC; *n* = 3) that included granulomatous lesions, ulcerations and gross structural damage to the colon.

**Table 1:** Clinical characteristics of African AIDS patients with unexplained diarrhea and/or weight loss

Age	Mean ± S.D.	35 ± 11
	Median (Range)	33 (21–52)
Gender	Male	12 (52 %)
	Female	11 (48 %)
CD4 count (cells/μl)	Mean ± S.D.	90 ± 68
	Median (Range)	69 (6–237)
% CD4 cells	Mean ± S.D.	8.5 ± 5.5
	Median (Range)	7.4 (0.42–22.7)
log_10_ HIV RNA copies/ml	Mean ± S.D.	4.89 ± 0.97
	Median (Range)	5.08 (4.2–6.6)
GIT complications	Diarrhea	13 (56 %)
	Weight loss	10 (44 %)

### The colon of AIDS patients with unexplained diarrhea and/or wasting contains inflammatory cell infiltrates and increased levels of pro-inflammatory cytokines

**Fig. 1:**
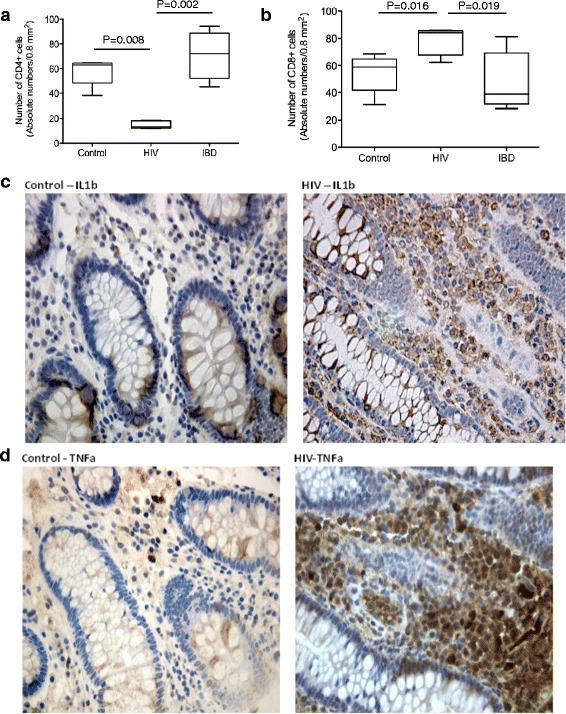
Inflammation in the colon of AIDS patients occurs in the setting of severe T cell dysregulation. **a** Differences in the frequency of CD4^+^ T in the colon of AIDS patients (*n* = 23) relative to uninfected healthy controls (*n* = 5) and IBD patients (*n* = 5) as determined by IHC. **b** Increased number of CD8^+^ T cells in the colon of AIDS patients relative to controls (*p* = 0.016) and patients with IBD (*p* = 0.019), as determined by IHC. Graphs show the median values and statistical significance between groups as calculated by Kruskal-Wallis with Dunn’s post-test. **c** Representative IHC staining showing increased levels of IL-1β in the colon of AIDS patients vs. uninfected controls (*p* = 0.04). **d** IHC staining showing increased TNF-α expression in the colon of AIDS patients compared to controls (*p* = 0.01)

Understanding the potential contribution of intestinal macrophages to HIV-induced pathogenesis requires a comprehensive analysis of the local microenvironment. The current study was performed on a relatively homogeneous population of co-pathogen negative ART-naïve patients with late-stage disease. As in previous studies [40], there was a dramatic reduction in the number CD4^+^ T cells and a corresponding increase in CD8^+^ T cell numbers in the colon of AIDS compared to HIV-negative IBD patients and healthy controls (CD4 counts: median of 13 vs. 72 vs. 63 cells/0.8 mm^2^, respectively; CD8 counts: median of 84 vs. 39 vs. 59 cells/0.8 mm^2^ respectively) (Fig. 1a and b). Mild-to-moderate non-specific colitis, as measured by the infiltration of inflammatory cells, namely CD8^+^ T cells, eosinophils and plasma cells, was detected in the colon of AIDS patients. IHC staining indicated that there was a marked increase in proinflammatory cells expressing IL-1β (median 174 vs. 75 cells/0.8 mm^2^, *p* = 0.04) and TNF-α (median 226 vs. 107 cells/0.8 mm^2^, *p* = 0.01) in the colon of AIDS patients compared to healthy controls (Fig. 1c and d). Thus, diarrhea and wasting were associated with increased recruitment of inflammatory cells and increased pro-inflammatory cytokine production, even in the absence of overt OIs.

### Genes involved in macrophage activation and Th1 responses are preferentially up-regulated in the colon of AIDS patients

We next assessed changes in mRNA expression in total colonic tissue isolated from AIDS patients (*n* = 10), HIV-negative IBD patients (*n* = 6) and HIV-negative healthy controls (*n* = 5). Out of 84 genes tested using the Human Th17 for Autoimmunity and Inflammation PCR array (SABioscience), 23 were abnormally expressed in the colon of AIDS and/or IBD patients (Table 2). IL-18, CCL2, TRAF6 and IL-12Rβ1 were upregulated in both AIDS and IBD patients compared to controls. Consistent with IHC results, there was a significant increase in TNF-α (FC 7.11, *p* = 0.05) and IL-1β (FC = 4.27, *p* = 0.05) mRNA in AIDS patients compared to controls. HIV infection was also associated with increased expression of genes involved in chemotaxis and activation of monocytes/macrophages (CCL2 and TRAF6) and Th1 responses (IFN-γ and TBX21) as reported in Table 2. Genes associated with maintaining tissue homeostasis and an anti-inflammatory environment (ie. IL-10, TGF-β1 and suppressor of cytokine signaling (SOCS)3) were unchanged in AIDS patients, but increased in HIV-negative subjects with IBD, a finding that is suggestive of a tissue-protective response in these patients (Table 2). Thus, chronic diarrhea and/or weight loss in African AIDS patients is associated with increased levels of macrophage related cytokines (TNF-α and IL-1β) and chemokines (CCL2) compared to HIV-negative healthy controls.

**Table 2:** Fold changes in mRNA levels of immune mediators altered in African AIDS patients and in patients with IBD

		HIV vs. Controls		CD vs. Controls		UC vs. Controls	
		FC	*p*-value	FC	*p*-value	FC	*p*-value
Cytokines and Chemokines	IFN-γ	5.31	0.001	4.81	0.037	1.02	0.667
	TNF-α	7.11	0.005	14.27	0.001	13.65	0.001
	IL-1β	4.27	0.050	12.97	0.032	59.26	0.049
	IL-8	7.24	0.212	36.84	0.075	194.5	0.002
	IL-10	3.03	0.376	4.19	0.123	5.44	0.012
	IL-18	26.85	0.031	32.17	0.004	28.57	0.001
	TGF-β1	2.55	0.542	3.46	0.029	4.04	0.045
	CCL2	10.78	0.008	16.07	0.007	59.33	0.009
	CCL22	1.40	0.697	3.61	0.043	1.94	0.491
	CXCL2	1.77	0.387	2.25	0.447	3.23	0.097
Receptors	ICAM1	4.31	0.231	14.17	0.008	31.75	0.001
	IL12Rβ1	3.69	0.017	4.06	0.026	3.42	0.045
	TLR4	3.28	0.192	5.05	0.211	6.16	0.042
	IL6R	1.09	0.782	1.14	0.541	0.66	0.037
	IL17RE	0.64	0.521	0.67	0.245	0.38	0.041
	IL17Rβ	0.76	0.469	0.27	0.037	0.15	0.006
Transcription Factors	TRAF6	14.75	0.014	12.77	0.027	12.81	0.049
	SOCS1	2.29	0.512	4.24	0.076	8.27	0.029
	SOCS3	1.96	0.341	5.52	0.023	20.31	0.005
	S1PR1	3.12	0.231	4.29	0.049	7.15	0.009
	TBX21	5.73	0.012	4.04	0.038	2.53	0.234
	YY1	11.91	0.046	5.84	0.071	7.97	0.001
	CLEC7	2.09	0.432	2.49	0.381	4.84	0.043

*IFN-γ* Interferon-γ, *TNF-α* Tumour Necrosis Factor-α, *IL* Interleukin, *TGF-β1* Transforming Growth Factor-β1, *CCL* Chemokine (C-C motif) ligand, *CXCL* chemokine (C-X-C motif) ligand, *TLR* Toll-like receptors, *SOCS* suppressor of cytokine signaling proteins, *TRAF* TNF receptor associated factor, *TBX* T-box transcription factor, *YY* Yin Yang, *CLEC* C-type lectin domain family

### Macrophages in the colon of AIDS patients produce TNF-α and IL-1β and have an activated CD14^*+*^ phenotype

**Fig. 2:**
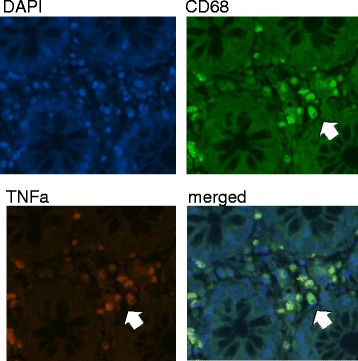
Macrophages in the colon of AIDS patients with unexplained diarrhea and/or weight loss produce TNF-α. Fluorescence microscopy of colon tissue from ART-naive AIDS patients dually stained with anti-CD68 (green) and anti-TNF-α (red) mAbs showing TNF-α^+^CD68^+^ macrophages in the *lamina propria*

**Fig. 3:**
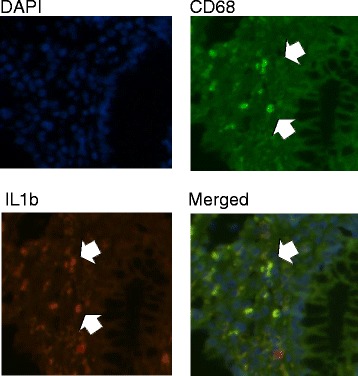
Macrophages in the colon of AIDS patients with unexplained diarrhea and/or weight loss produce IL-1β. Fluorescence microscopy of colon tissue from ART-naive AIDS patients dually stained with anti-CD68 (green) and anti-IL-1β (red) mAbs showing IL-1β^+^CD68^+^ macrophages in the *lamina propria*

**Fig. 4:**
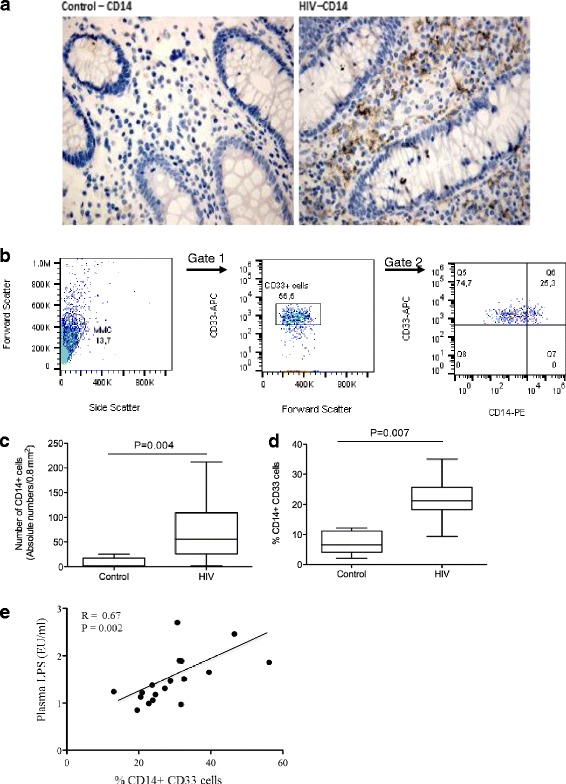
CD14^+^ macrophages accumulate in the colon of AIDS patients despite cotrimoxazole prophylaxis and absence of co-infections. **a** Representative IHC staining showing higher levels of CD14 expression on colonic macrophages of AIDS patients vs. uninfected healthy controls. **b** Representative histogram from flow cytometric analysis showing gating strategy and co-expression of CD14 in CD33+ cells. **c** Quantification of IHC analysis showing increased levels of CD14^+^ cells in the colon of AIDS patients relative to uninfected healthy controls; the median values and the statistical significance of the analysis between the two groups (*p* = 0.004) are indicated. **d** Summary of flow cytometric analyses showing increased expression of CD14 on CD33^+^ macrophages isolated from the colon of AIDS patients vs. uninfected healthy controls; the median values and the statistical significance of the analysis between the two groups are as indicated (*p* = 0.007). **e** Spearman correlations (R and p values) showing positive correlation between CD14^+^ macrophages in the colon of these patients and plasma levels of LPS (right panel). Results were considered significant if *p* < 0.05. Correlations were considered strong if r >0.6

To determine whether macrophages were contributing to the pro-inflammatory milieu in the colon of AIDS patients, we performed double labeling of CD68^+^ macrophages with TNF-α and IL-1β using a dye swap method with validation by fluorescence microscopy [26]. TNF-α^+^ and IL-1β^+^ CD68-positive macrophages were present in the *lamina propria* of AIDS patients (Figs. 2 and 3). As in CD, there was a marked increase in CD14^+^ macrophages in the colon of AIDS patients compared to HIV-negative controls. The absolute number of CD14^+^ cells, as determined by IHC, increased from 1.8 cells/HPF in controls to 55 cells/HPF (*p* = 0.004) in patients with AIDS; the percentage of CD14^+^ cells, as assessed by flow cytometry using CD33, increased from a median 6.5 to 21.2 % (*p* =0.007) (Fig. 4). In addition to CD14, CD33^+^ macrophages in AIDS patients co-expressed the Fc receptor γ (CD16) and T cell the co-stimulatory molecules CD80 and CD86 (Table 3). Of considerable interest was the detection of a positive correlation between the percentage of CD14^+^CD33^+^ colonic macrophages and the levels of plasma LPS (*R* = 0.67, *p* = 0.002). The percentage of CD14^+^CD33^+^ colonic macrophages did not correlate with any other clinical variable or marker of inflammation (e.g., plasma or tissue HIV viral load, CD4 count, age). Collectively, these findings indicate that macrophages in the colon of African AIDS patients with diarrhea and/or weight loss have an activated CD14^+^ phenotype and express pro-inflammatory cytokines (TNF-α, IL-1β) that are known to be induced by LPS–mediated activation of CD14^+^ macrophages [41].

**Table 3:** Phenotypic characterization of colonic macrophages as determined by flow cytometry

	CD14^+^ (median % of CD33^+^ cells)	CD16^+^ (median % of CD33^+^ cells)	CD80^+^ (median % of CD33^+^ cells)	CD86^+^ (median % of CD33^+^ cells)
HIV^−^	6.5 ± 4.8	11.1 ± 7.6	9.2 ± 5.5	15.1 ± 7.3
HIV^+^	21.2 ± 11.7	19.4 ± 12.5	25.1 ± 15.4	23.7 ± 18.1

### LPS stimulation induces increased production of macrophage related pro-inflammatory cytokines in MMC isolated from the colon of AIDS patients

**Fig. 5:**
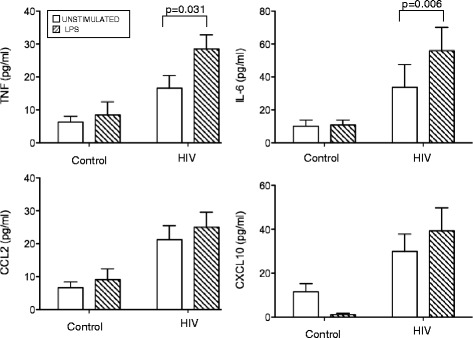
LPS induces increased pro-inflammatory cytokine production by MMC isolated from the colon of AIDS patients. Total MMC isolated from the colon of African AIDS patients (*n* = 10) and healthy uninfected healthy controls (*n* = 5) were cultured for 18 h in the presence or absence of LPS. Cytokine and chemokine levels in the culture supernatants were quantified using customized Bio-Plex plates from BioRad. The results represent the mean ± SD of two independent experiments performed in triplicate. Statistical significance between groups was calculated using the Mann–Whitney *U* test. Statistically significant differences (*p* < 0.05) are indicated by an asterisk

**Fig. 6:**
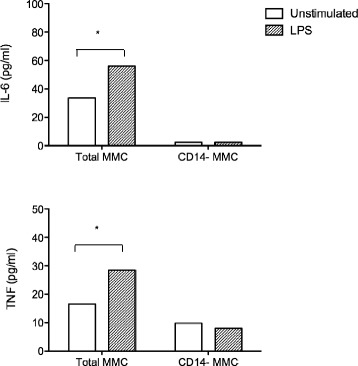
Depletion of CD14^+^ macrophages leads to a marked decrease in the ability of MMC to produce TNF and IL-6, both constitutively and in response to LPS stimulation. CD14^+^ macrophages were depleted from MMC isolated from the colon of ART-naïve AIDS patients. Total (CD14^+^) and CD14-depleted MMC were then cultured for 18 h in the presence or absence of LPS. Cytokine/chemokine secretion into the culture supernatant was quantified using customized Bio-Plex plates. Results represent the mean ± SD of triplicate assays performed on 10 African patients with AIDS. Statistical significance between groups was calculated using the Mann–Whitney *U* test. Statistically significant differences (*p* < 0.05) are indicated by an asterisk

To assess the LPS responsiveness of colonic immune cells, total and CD14-depleted MMC isolated from the colon of 10 HIV^+^ patients and 5 healthy controls were cultured in vitro for 18 h in the presence or absence of LPS. Secretion of cytokines and chemokines into the culture supernatant was measured using customized Bio-Plex plates. In the absence of LPS, unstimulated total MMC from AIDS patients secreted significantly higher levels of macrophage related pro-inflammatory cytokines (TNF-α and IL-6) and chemokines (CCL2, CXCL10) compared to the MMC isolated from healthy controls (Fig. 5). LPS stimulation of total MMC from AIDS patients induced a further increase in TNF-α (median: 17.9 vs 31.8 pg/mL in unstimulated vs. stimulated MMC, respectively, *p* = 0.031) and IL-6 (median: 19.3 vs 45.46 pg/mL, respectively, *p* = 0.006) secretion. In contrast, LPS failed to increase CCL2 and CXCL10 secretion in MMC, suggesting that the upregulation of these chemokines is controlled by an LPS-independent mechanism. When MMC from AIDS patients were depleted of CD14^+^ cells (ie. CD14^−^ MMC), they produced less TNF-α (median: 9.7 pg/ml, *p* = 0.032) and IL-6 (median 2.2 pg/mL, *p* = 0.002) than unfractionated MMC, suggesting that CD14^+^ macrophages played a major role in the expression of these pro-inflammatory cytokines (Fig. 6). Unlike CD14^+^ MMC, LPS had no inductive effect on cytokine production in CD14-depleted MMC. These results suggest that CD14^+^ macrophages in the colon of African AIDS patients presenting with diarrhea and/or weight loss may have increased responsiveness to microbial products, a finding that would be expected to perpetuate the local inflammatory response.

## Discussion

In this study, we used several different in situ (IHC) and ex vivo (flow cytometry, cytokine/chemokine production, mRNA profiling) methods to assess the phenotype and functional parameters of macrophages in the colon of ART-naïve African AIDS patients with unexplained gastrointestinal symptoms. Despite prophylactic treatment with cotrimoxazole and the apparent absence of OIs, we found that the colon of AIDS patients with chronic diarrhea and/or weight loss was highly inflamed and contained increased numbers of inflammatory cell infiltrates. The total expression of cytokines, chemokines and transcription factors associated with macrophage recruitment and activation was also significantly increased in the colon of AIDS patients compared to negative controls. The pattern of cytokine/chemokine upregulation in these patients was similar to that observed in HIV-negative subjects with CD. In addition, we detected a significant increase in activated CD14^+^ macrophages that produced pro-inflammatory cytokines and were responsive to LPS stimulation supporting the view that, as in CD [30, 32, 36], disrupted or abnormal differentiation of macrophages may be a major cause of the excessive inflammation and loss of tolerance to commensal bacteria that is observed in HIV-infected patients. While we did not observe any direct associations between the levels of CD14+ macrophages and tissue or plasma HIV viral loads, we speculate that high levels of HIV replication in the gastrointestinal tract during acute infection results in increased the recruitment of inflammatory monocytes. Unlike resident tissue macrophages, these newly recruited cells differentiate into macrophages that produce pro-inflammatory cytokines and chemokines in response to bacterial and viral products. Production of these pro-inflammatory mediators, together with residual viral replication and ongoing recruitment and differentiation of monocytes into pro-inflammatory macrophages, leads to further propagation of the inflammatory response a process that continues throughout the course of infection.

In the current study, we observed increased recruitment of inflammatory cell infiltrates (CD8^+^ T cells, eosinophils and/or plasma cells) and increased production of pro-inflammatory cytokines (including TNF-α and IL-1β) in the colon of ART-naive patients with late-stage HIV infection. At the mRNA level, we detected a significant increase in transcripts coding for pro-inflammatory cytokines and their receptors (TNF-α, IL-1β, IL-18, IFN-γ, IL-12RB1), chemokines (CCL2, CXCL10) and inflammation-related transcription factors (TRAF6, TXB21, YY1) in AIDS patients compared to HIV-negative controls. These findings are consistent with North American and European studies showing increased expression of pro-inflammatory cytokines in the rectosigmoid colon and duodenum of ART-naïve patients [39, 42, 43] with peak expression of TNF-α and IL-1β mRNA in the rectum occurring in patients with AIDS [44]. Our data is also consistent with studies showing increased expression of chemokines (CCL5, CXCL9, and CXCL10) in the duodenum of untreated HIV-infected patients [39]. Many of the immune mediators expressed at high levels in AIDS patients were also up-regulated in patients with CD (TNF-α, IL-1β, and IFN-γ) and have been implicated in the pathogenesis of CD [45]. As observed for HIV, CD is associated with systemic inflammation and increased plasma levels of markers of microbial translocation [46–48]. In CD, these changes are associated with increased secretion of IL-12 by activated intestinal macrophages. Higher levels of IL-12 drive increased production of IFN-γ in T cells, increased epithelial permeability and further enhancement of macrophage activation and microbial translocation [29]. Given the marked increase in IFN-γ observed in the colon of AIDS patients, it is conceivable that a similar feedback loop may be occurring in ART-naïve patients with advanced HIV infection.

Phenotypic and functional characterization of intestinal macrophages indicated that there was marked a increase in a unique subset of pro-inflammatory (CD14^+^) mononuclear phagocytes in the colon of cotrimoxazole-treated ART-naïve AIDS patients compared to uninfected controls. As in CD, these mononuclear phagocytes produced TNF-α and IL-1β and expressed innate response receptors including CD14, the co-receptor for LPS. Although these cells are believed to be macrophages based on their expression of CD33 and CD68, we cannot exclude the possibility that they may also have some properties of myeloid dendritic cell (mDC). Studies by Kamada et al. have reported that CD14^+^CD33^+^CD68^+^ “macrophages” in the colon of CD patients co-express the mDC markers, CD205 and CD209 (DC-SIGN) [29, 30].

Ex vivo studies of total MMC (containing CD14^+^ macrophages) isolated from the colon of AIDS patients indicated that these cells secreted increased levels of TNF-α, IL-6, CCL2 and CXCL10 compared to HIV-negative controls. Removal of CD14^+^ cells resulted in a marked decrease in the ability of MMC to produce pro-inflammatory cytokines suggesting that CD14^+^ macrophages are an important source of pro-inflammatory mediators in ART-naïve patients with late-stage HIV infection. Macrophages isolated from AIDS patients also had an increased ability to respond to microbial products including LPS, a TLR4 ligand, presumably due to the upregulation of CD14 and other innate response receptors. The positive correlation observed between CD14^+^ macrophages and plasma LPS suggests that the increased frequency of these cells in the colon of AIDS patients is linked to MT. Additional studies will be required to determine whether CD14^+^ macrophages are responsive to TLR-ligands other than LPS and to better define the relationship between CD14^+^ macrophages and microbial translocation. Studies are also needed to assess the phagocytic properties of CD14^+^ macrophages in the colon of African AIDS patients. A recent study by Allers et al., has shown that mononuclear cells isolated from the duodenum of HIV-infected patients have a reduced ability to phagocytose *E coli* BioParticles and thus, may be less likely to eliminate antigenic and microbial products that have crossed the epithelial barrier [39].

The strengths of our study relate to the homogeneity of our patient cohort with respect to treatment, stage of infection, viral load and CD4^+^ T cell counts. Limitations relate to the small size of our positive (IBD) and negative control groups. In addition, despite efforts to exclude patients with enteric co-infections (cotrimoxazole prophylaxis, stool culture and histological staining for acid-fast bacilli, fungal and parasitic infections), we cannot rule out the possibility that opportunistic co-infections may have affected our results. Additional studies are needed to determine whether our results can be extended to patients with early HIV infection and ART-treated patients with and without enteric co-infections. Studies exploring the relationships between increased inflammatory macrophage infiltrate and alterations in the functional gene content of intestinal microbiota are also of major importance given the vital role of the microbiota in shaping the intestinal microenvironment.

## Conclusions

We have demonstrated that the colon of cotrimoxazole-treated ART-naïve AIDS patients contains increased numbers of activated CD14^+^ pro-inflammatory macrophages and that these cells are responsive to bacterial LPS and linked to MT. This study underscores the similarities between HIV and non-infectious IBD and highlights the need for detailed comparative studies to determine if there is a common mechanism driving these inflammatory pathologies. It will be especially important to determine whether the persistence of inflammation and immune activation in cotrimoxazole-treated patients is driven by HIV replication or by alterations in the intestinal microenvironment (such as changes in the microbiome [49, 50]; irreversible (or reversible) damage to the intestinal epithelium and/or non-specific activation of CD8^+^ T cells). This information is needed to design highly targeted treatments that can restore the integrity of the intestinal epithelium, normalize the intestinal microbiota and prevent MT.

## Abbreviations

6-MP: 6-Mercaptopurine
Ab: Antibody
AFB: Acid fast bacilli
AIDS: Acquired Immune Deficiency Syndrome
ART: Antiretroviral therapy
CCL: Chemokine (C-C motif) ligand
CD: Crohn’s disease
CXCL: Chemokine (C-X-C motif) ligand
DAB: 3,3′-Diaminobenzidine
EDTA: Ethylenediaminetetraacetic acid
FCS: Fetal calf serum
GIT: Gastrointestinal tract
H&E: Hematoxylin and eosin
HIV: Human Immunodeficiency Virus
HPR: High power fields
HRP: Horseradish peroxidase
IBD: Inflammatory Bowel Disease
IHC: Immuno-histochemistry
IFN: Interferon
IL: Interleukin
LPS: Lipopolysaccharide
mAbs: Monoclonal antibodie
mDC: Myeloid dendritic cells
MMC: Mucosal mononuclear cells
MT: Microbial translocation
OIs: Opportunistic infections
PAS-d: Periodic acid-Schiff diastase
RPMI: Roswell Park Memorial Institute medium
SOCS: Suppressor of cytokine signaling
TBX: T-box
TGF: Transforming growth factor
Th17: T helper 17 cell
TLR: Toll like receptor
TNF: Tumour necrosis factor
TRAF: TNF receptor-associated factor
Treg: T regulatory cells
UC: Ulcerative colitis
VL: Plasma viral load
